# Etanercept, a Widely Used Inhibitor of Tumor Necrosis Factor-α (TNF- α), Prevents Retinal Ganglion Cell Loss in a Rat Model of Glaucoma

**DOI:** 10.1371/journal.pone.0040065

**Published:** 2012-07-03

**Authors:** Miin Roh, Yan Zhang, Yusuke Murakami, Aristomenis Thanos, Sung Chul Lee, Demetrios G. Vavvas, Larry I. Benowitz, Joan W. Miller

**Affiliations:** 1 Angiogenesis Laboratory and Retina Service, Department of Ophthalmology, Massachusetts Eye and Ear Infirmary, Harvard Medical School, Boston, Massachusetts, United States of America; 2 Department of Ophthalmology, Institute of Vision Research, Yonsei University College of Medicine, Seoul, Korea; 3 Laboratories for Neuroscience Research in Neurosurgery and F. M. Kirby Neurobiology Center, Children’s Hospital, Departments of Surgery and Ophthalmology and Program in Neuroscience, Harvard Medical School, Boston, Massachusetts, United States of America; Dalhousie University, Canada

## Abstract

**Background:**

Visual loss in glaucoma is associated with pathological changes in retinal ganglion cell (RGC) axons and a slow decline in the RGC population. Age and elevated intraocular pressure (IOP) are the main risk factors for glaucomatous loss of vision. Several studies have implicated the proinflammatory cytokine tumor necrosis factor- α (TNF-α) as a link between elevated IOP and RGC death, but the cellular source of TNF-α and its causative role in RGC death remain uncertain. Here, using a rat model of glaucoma, we investigated the source of elevated TNF- α and examined whether Etanercept, a TNF-α blocker that is in common clinical use for other indications, is protective against RGC death.

**Methodology/Principal Findings:**

Episcleral vein cauterization (EVC) caused intraocular pressure (IOP) to be elevated for at least 28 days. IOP elevation resulted in a dramatic increase in TNF-α levels within a few days, axonal degeneration, and a 38% loss of RGCs by 4 weeks. Immunostaining coupled with confocal microscopy showed that OHT induced robust induction of TNF-α in Iba-1-positive microglia around the optic nerve head (ONH). Despite persistent elevation of IOP, Etanercept reduced microglial activation, TNF-α levels, axon degeneration in the optic nerve, and the loss of RGCs.

**Conclusions/Significance:**

Ocular hypertension (OHT) triggers an inflammatory response characterized by the appearance of activated microglia around the ONH that express TNF-α. Blocking TNF-α activity with a clinically approved agent inhibits this microglial response and prevents axonal degeneration and loss of RGCs. These findings suggest a new treatment strategy for glaucoma using TNF- α antagonists or suppressors of inflammation.

## Introduction

Retinal ganglion cell (RGC) death and subsequent visual field defects that progress to blindness are the underlying pathophysiology of glaucoma [1]. Age is the leading risk factor, with elevated intraocular pressure (IOP) being the only risk factor that can be modified [2]–[4]. Lowering IOP with surgery or drugs reduces the rate of optic nerve head (ONH) damage and progressive visual field loss by almost half, firmly establishing IOP reduction as an effective treatment for glaucoma. Proposed mechanisms linking RGC loss to elevated IOP include a compressive effect on the cribriform plates of the lamina cribrosa [5], pressure-induced tissue ischemia [6], [7], and local cellular response mechanisms [8].

Considerable evidence suggests that the damage begins within the optic nerve due to structural changes within the lamina cribrosa [9], leading to cellular changes that influence RGC viability [10]. Histopathological studies of the glaucomatous ONH reveal astrocyte and microglial activation accompanying neural damage [11], [12]. Activated microglia display an altered morphology, producing cytotoxic and degenerative factors [13], [14].

TNF-α is a proinflammatory cytokine that is secreted in response to infection and trauma, and can lead to apoptosis in susceptible cells through the activation of caspases [15] or indirectly via activation of microglia [16]. TNF-α and its receptor have been detected in the ONH of glaucoma patients [12], [17], [18] and in a rat model of glaucoma [19], suggesting that TNF-α may be an important factor in the neurodegenerative process of glaucoma. Using a mouse model of glaucoma, we previously found that TNF-α mediates the cytotoxic effect of ocular hypertension (OHT) on RGCs through a mechanism that involves microglial activation and loss of oligodendrocytes [20]. However, those studies left open several questions, including the cellular source of TNF-α, whether the observed RGC loss was due to the particular method of OHT induction that was used, whether the findings would generalize to other species, and whether RGC loss could be attenuated using clinically available treatments. Etanercept (Enbrel®) is a decoy receptor consisting of the ligand-binding domain of the TNF type II receptor and the Fc component of human immunoglobulin G1. Etanercept competitively inhibits the binding of free TNF-α and TNF-β to cell surface receptors, and is used clinically for rheumatoid arthritis, juvenile idiopathic arthritis, ankylosing spondylitis, and psoriatic arthritis [21], [22]. In rats with endotoxin-induced uveitis, subcutaneous injection of Etanercept reduced the level of TNF-α and decreased intraocular inflammation [23]. The aims in the present study were to examine the expression of TNF-α in a rat model of chronic OHT, determine the cellular localization of TNF-α, and evaluate whether Etanercept would decrease TNF-α levels and reduce optic nerve degeneration and RGC loss.

## Results

### Systemic Treatment with Etanercept does not Affect Intraocular Pressure

**Figure 1:**
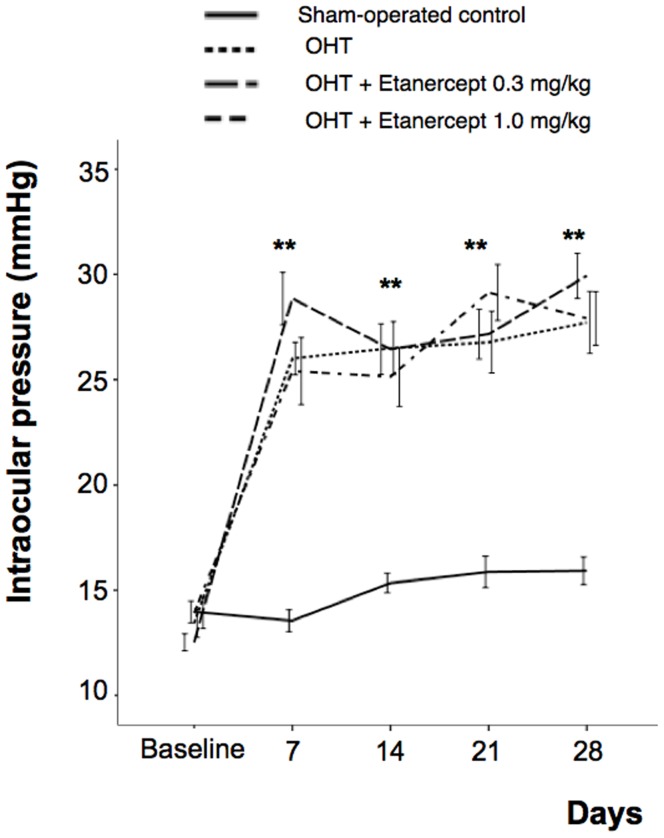
Etanercept does not affect intraocular pressure (IOP) after episcleral vein cauterization (EVC). EVC increases IOP over a 4-week period, and Etanercept does not block this increase at either 0.3 or 1.0 mg/kg (n = 10 rats for each time point).

We induced OHT in the right eyes of rats (n = 40) by cauterizing the episcleral vein, leaving the left eye as a control. Whereas the average IOP in the control eye was 14.4±0.3 mm Hg, IOP rose to 47.6±12.7 mm Hg immediately after cauterization and remained elevated for the duration of the study in 80% (n = 32) of the eyes at 4 weeks after EVC; 12.5% (n = 5) fell into phthisis and 7.5% (n = 3) did not meet the criteria for successful OHT induction. In the OHT groups, Etanercept (0.3 or 1.0 mg/kg) or saline was injected intraperitoneally three times a week from the time of initial IOP elevation until the time of euthanasia. Over the next 4 weeks, IOP remained stable at approximately 2.2-fold above baseline in the group treated with 0.3 mg/kg Etanercept, 1.9 -fold in the group treated with 1.0 mg/kg Etanercept, and 2-fold in the sham-operated control group; these values did not differ from one another (Kruskal-Wallis test, all P>0.05 at each time point: Fig. 1). Thus intraperitoneal injection of Etanercept had no effect on IOP.

### Detection of Etanercept in the Eye

Etanercept is a recombinant chimeric protein generated by fusing the ligand-binding portion of human TNF- α receptor TNFR-2 and the Fc portion of human IgG1. To investigate whether intraperitoneally injected Etanercept reaches the retina, we immunoprecipitated retinal lysates treated with Etanercept or normal saline with an antibody against human IgG1. The immunoprecipitate from animals treated with Etanercept contained one strong band at the appropriate size that was absent in immunoprecipitates from mice receiving normal saline (Fig. S1).

### TNF-α Levels Increase with OHT

**Figure 2:**
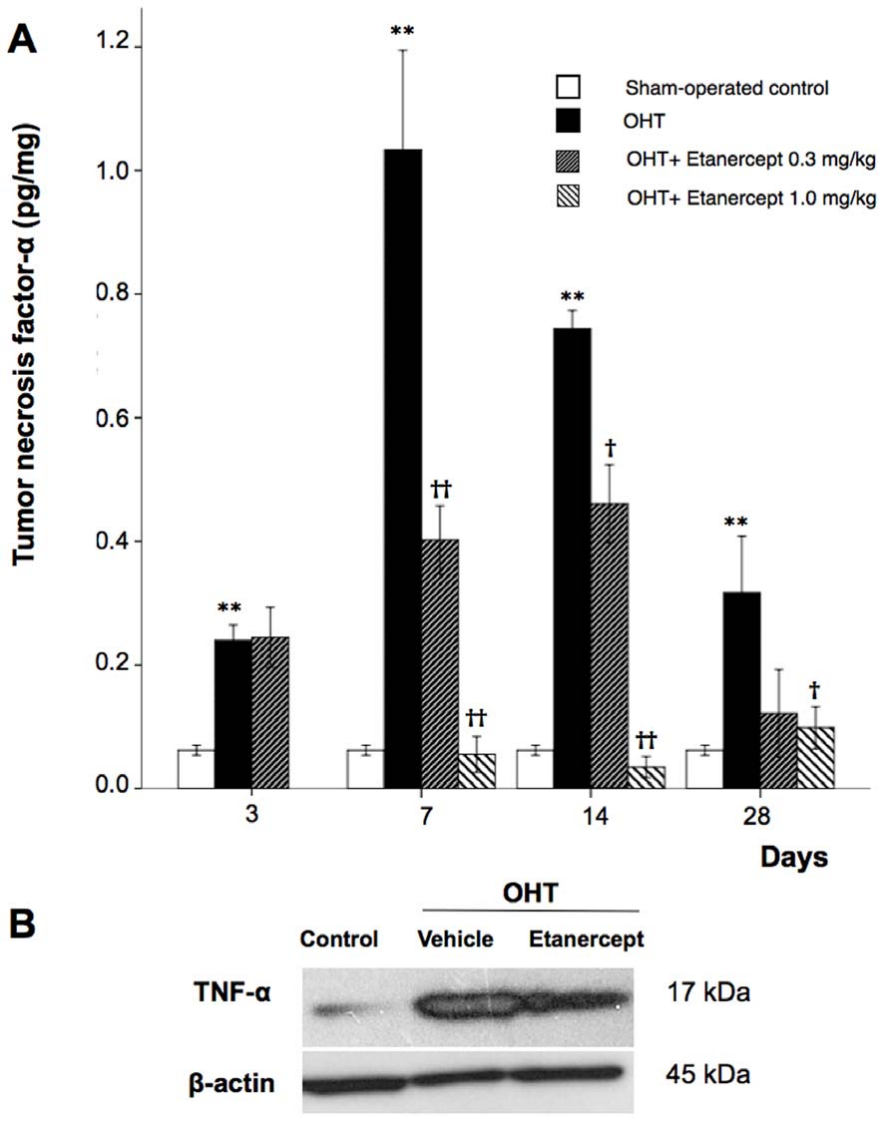
Etanercept suppresses TNF-α elevation following IOP elevation. **A**, ELISA for TNF-α protein in the retina. Mann-Whitney U test, *P<0.05, **P<0.01 comparing sham-operated control and vehicle-treated OHT; ^†^P<0.05, ^††^P<0.01 comparing OHT rats treated with vehicle vs. Etanercept (n = 6 for each group at each time point). **B**, Western blot analysis of TNF-α 14 days after OHT induction with and without Etanercept (n = 4 for each group). OHT, Ocular hypertension; Etan., Etanercept.

Previous studies have reported elevated levels of TNF-α in the retina and ONH of glaucoma patients [11], [12], [17], [24]. We investigated whether this elevation could be detected in the present rat model by measuring TNF-α levels by ELISA in retinal lysates. The ELISA results are normalized by the level of protein per retina. ELISA showed that the level of TNF-α increased 3.9-fold three days after OHT induction relative to sham-operated control rats and continued to rise. Seven days after OHT induction, TNF-α levels were 1.0±0.5 pg/mg, approximately 17 times higher than in sham-operated control rats. Levels declined thereafter but remained elevated for at least 4 weeks. Treatment with 0.3 mg/kg of Etanercept reduced the level of TNF-α 7 days after treatment initiation (Mann-Whitney U test, P = 0.001), and the higher dose (1.0 mg/kg) led to an even stronger reduction (Mann-Whitney U test, P<0.001: Fig. 2A). Western blot analysis confirmed the elevation of TNF-α 14 days after the induction of OHT and the reduction in TNF-α after Etanercept treatment (0.3 mg/kg, Fig. 2B).

### Cellular Source of TNF-α

**Figure 3:**
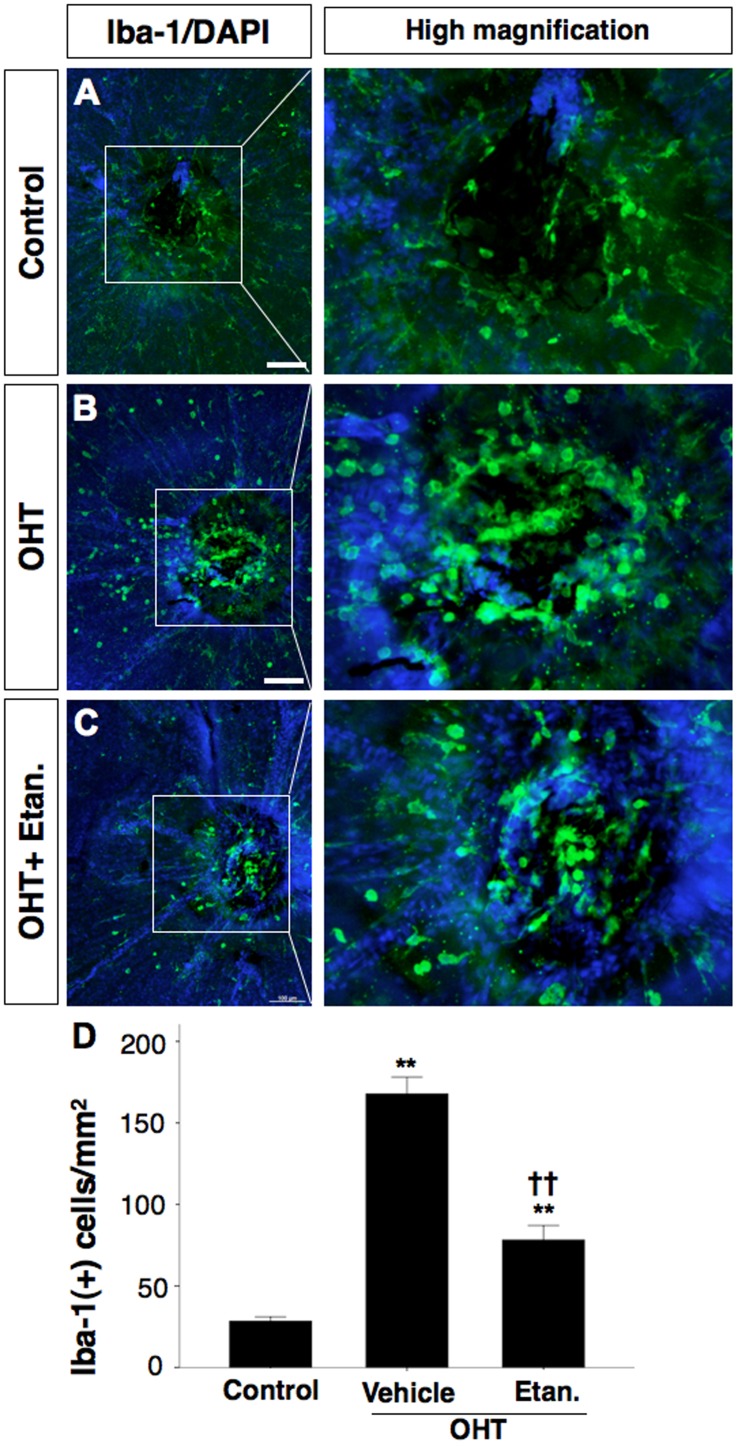
Activation of microglia in the vitreal surface of the optic nerve head (ONH). **A**, Iba-l positive microglia in sham-operated control eyes are ramified and quiescent and exhibit a mosaic arrangement around ONH. **B**, Increased number of activated microglia with short, broad processes 5 days after OHT induction. **C**, Decreased number of active microglia around the ONH with Etanercept treatment. **D**, Bar graph indicating the number of stage 3 and 4 Iba-1 positive microglia/mm^2^. Mann-Whitney U test, **P<0.01 comparing sham-operated control and OHT cases treated with vehicle or Etanercept; ^††^P<0.01 comparing OHT cases treated with vehicle vs. Etanercept. Data are expressed as the mean ± SEM (n = 6 for each group). Original magnification, ×8 (A–C); Scale bar, 100 µm. OHT, ocular hypertension; Etan., Etanercept 1.0 mg/kg.

To identify the source of TNF-α, we carried out immunostaining on retinal whole-mounts. Immunostaining with the Iba-1 antibody revealed differences in the appearance of microglia in the ONH area 5 days after OHT induction. Microglial activation is characterized by morphological changes such as swelling of the cell body, thickening of the proximal processes, and a reduction of distal ramifications [25]. In control eyes with sham surgery, most Iba-1-positive cells were stage 1 resting microglia, with very few stage 3 and 4 cells around the ONH (28.3±2.6 cells/mm^2^: Fig. 3A). With OHT, we observed increased numbers of stage 3 and 4 microglia with round-shaped cell bodies and thick, stout processes or no processes at all around the ONH (167.8±10.3 cells/mm^2^, Mann-Whitney U test, P<0.001: Fig. 3B). Etanercept treatment resulted in a 53% decrease in the number of infiltrative stage 3 and 4 microglia around the ONH compared to untreated OHT rats (78±9 cells/mm^2^, Mann-Whitney U test, P<0.001: Fig. 3C, D).

**Figure 4:**
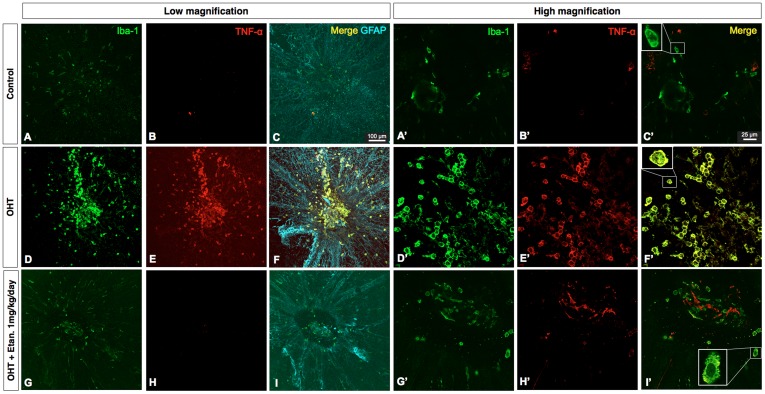
Confocal images of immunofluorescent staining for Iba-1 (green), TNF-α (red), and GFAP (cyan). Merged image demonstrates the colocalization of TNF-α and Iba-1 (C, F, I). Square insets show enlargements of selected cells (***C’, F’, and I’***). Original magnification: ×10 (A–H), ×63 (A′–H′)*;* Scale bars: 100 µm at lower magnification, 25 µm at higher magnification.

We also carried out confocal microscopy on tissue double-labeled with antibodies against microglia (Iba-1) and astrocytes (GFAP). In sham-operated (control) retinal whole-mounts, we observed resting microglia with ramified processes distributed in a mosaic pattern around the ONH, with very few amoeboid-shaped cells and minimal GFAP expression (Fig. 4C, C′). Five days after OHT induction, increased numbers of microglia were found around the ONH along with increased expression of the astrocyte marker, GFAP (Fig. 4F). Iba-1-positive microglia were mostly amoeboid-shaped and were double-stained with antibodies to TNF-α (Fig. 4F′). Etanercept prevented the infiltration of amoeboid-shaped microglia and GFP expression. In addition, few Iba-1-positive microglia showed TNF-α staining in Etanercept-treated eyes (Fig. 4I′).

### Etanercept Prevents Pathologic Changes in the Optic Nerve

**Figure 5:**
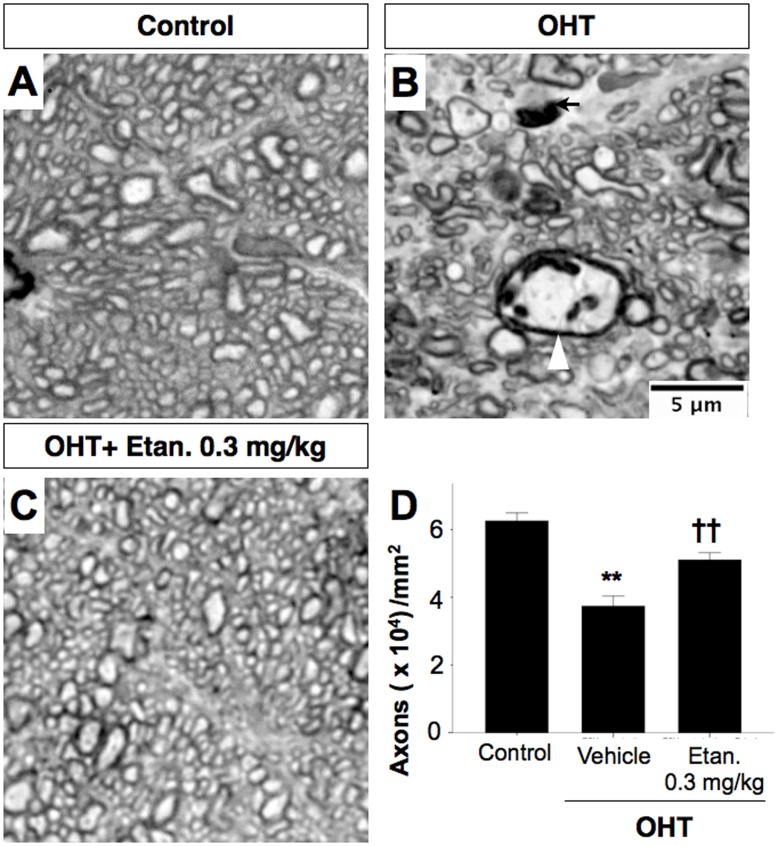
Etanercept prevents optic nerve degeneration after OHT induction. **A–C**, High magnification light micrographs of cross sections through rat optic nerve. EVC leads to diminished axon density, enlarged axons (*white arrowhead*), and degenerating axon profiles (*black arrow* in ***B***). **D**, Bar graph showing axon densities in the various groups. Data are expressed as the mean ± SEM. Mann-Whitney U test, **P<0.01 compared between sham-operated control and vehicle-treated OHT, ^††^P<0.01 comparing OHT cases treated with vehicle vs. Etanercept (n = 6 for each group). Original magnification: ×100; Scale bar, 5 µm. OHT, Ocular hypertension; Etan., Etanercept.

**Figure 6:**
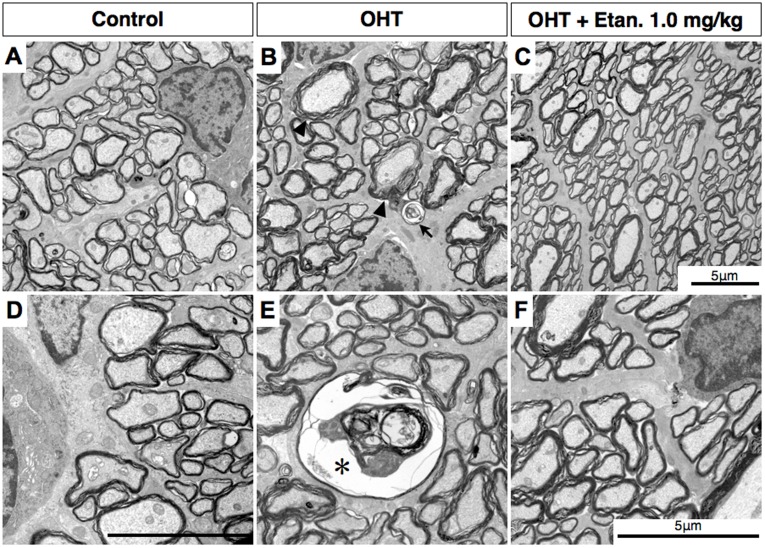
Etanercept prevents axon degeneration after IOP elevation. **A, D**, Electron micrograph showing normal compact axoplasm and myelination in sham-treated control optic nerve. **B, E**, Axonal swelling with separation of myelin sheath (*black arrowhead*), degraded collection of altered tubulovesicular structures (***E***, *asterisk*), and a whorl-shaped mass (***C***, *black arrow*). **C, F**, Normal-appearing axons in OHT rat treated with Etanercept. Original magnification: ×3400 (A–C), ×5800 (D–F); Scale bar, 5 µm. OHT, Ocular hypertension; Etan., Etanercept.

We next compared axon morphology and density in cross-sections through the optic nerve. After 28 days of elevated IOP, the nerve showed marked axonal disorder compared to sham-operated controls, with a heterogeneous caliber of myelinated axons, increased vacuolization, and ballooning (Fig. 5B). Etanercept prevented this degeneration, resulting in an appearance similar to that of the control nerves (Fig. 5C). In terms of total axons, the axon density in sham-operated control nerves was 62,553±12,966 axons/mm^2^, and this number decreased by 40.2% in OHT eyes (Mann-Whitney U test, P<0.001). Etanercept (0.3 mg/kg) maintained axon density at considerably higher levels than were seen in saline-treated controls (Mann-Whitney U test, P = 0.001: Fig. 4D) with only minor degenerative changes. We next examined the morphology of axons by transmission electron microscopy (TEM). The normal sham-operated optic nerve shows a compact arrangement of myelinated axons (Fig. 6A, D). With OHT, the major change was the appearance of swollen axons (Fig. 6B, arrowhead) with occasional cytoskeletal disintegration (Fig. 6E, *asterisk*) and loosely organized myelin with multilayered, whorled masses (Fig. 6B, arrow). Axons in the optic nerves of Etanercept-treated OHT rats appeared nearly normal (Fig. 6C, F).

### Neurofilament Expression Decreases with OHT

**Figure 7:**
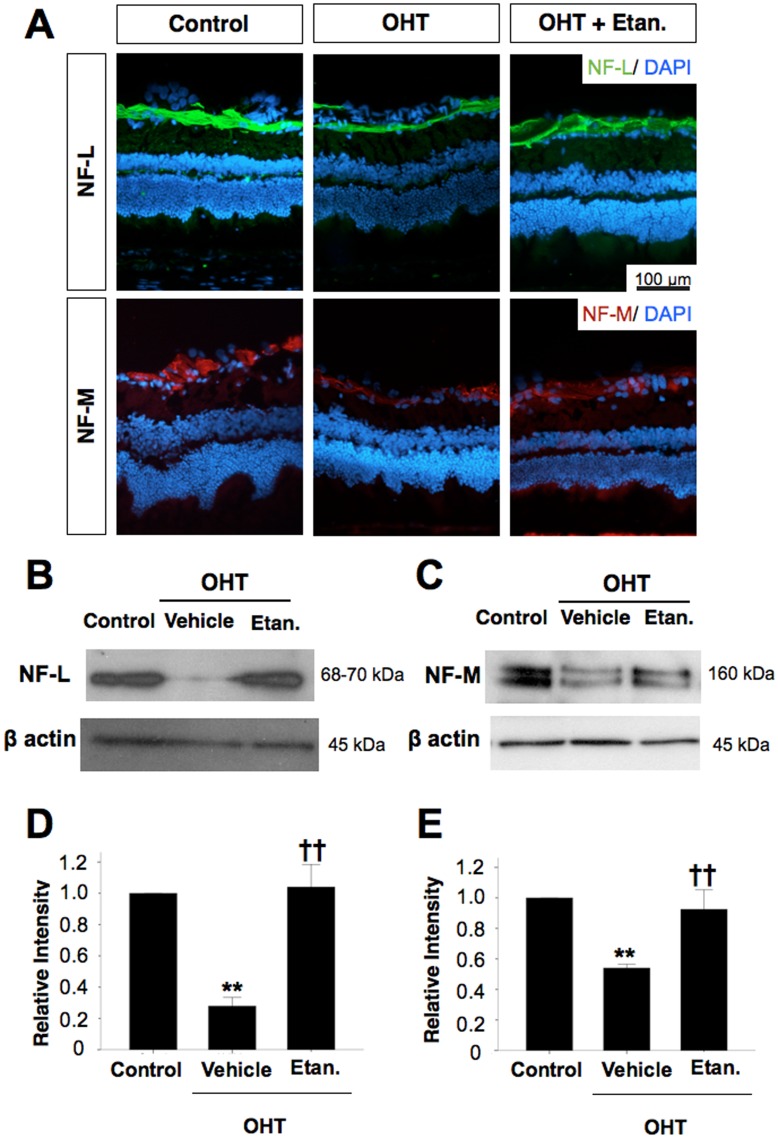
Etanercept prevents the loss of cytoskeletal proteins following OHT induction. **A**, Immunohistochemical staining for neurofilament light chain (NF-L) and middle chain (NF-M) proteins around the ganglion cell layer. Western blot analysis for NF-L (**B**) and NF-M (**C**) after OHT in rats treated with or without Etanercept (n = 4 in each lane). Differences in loading were normalized by the level of β-actin. Bar graphs indicating relative levels of NF-L (**D**) and NF-M (**E**) to β-actin by densitometry, reflecting the results from four independent experiments. Mann-Whitney U test, **P<0.01 comparing sham-operated control and vehicle-treated OHT; ^††^P<0.01 comparing OHT rats treated with vehicle vs. Etanercept. Original magnification : ×20, Scale bar, 100 µm. OHT, Ocular hypertension; Etan., Etanercept; NF-L, neurofilament-L; NF-M, neurofilament-M.

To examine changes in proteins associated with the axonal cytoskeleton, we performed western blot analysis and immunohistochemical staining to detect the light- and medium chains of neurofilaments (NF-L, NF-M, respectively). In the retina, both NF-L and NF-M antibodies stained axon fascicles around the ganglion cell layer (Fig. 7A). Protein analyses show that expression of both NF-L and NF-M decreased 14 days after induction of OHT, whereas Etanercept maintained the expression of both proteins close to normal levels (Fig. 7B–E).

### Etanercept Prevents Retinal Ganglion Cell Loss with OHT

**Figure 8:**
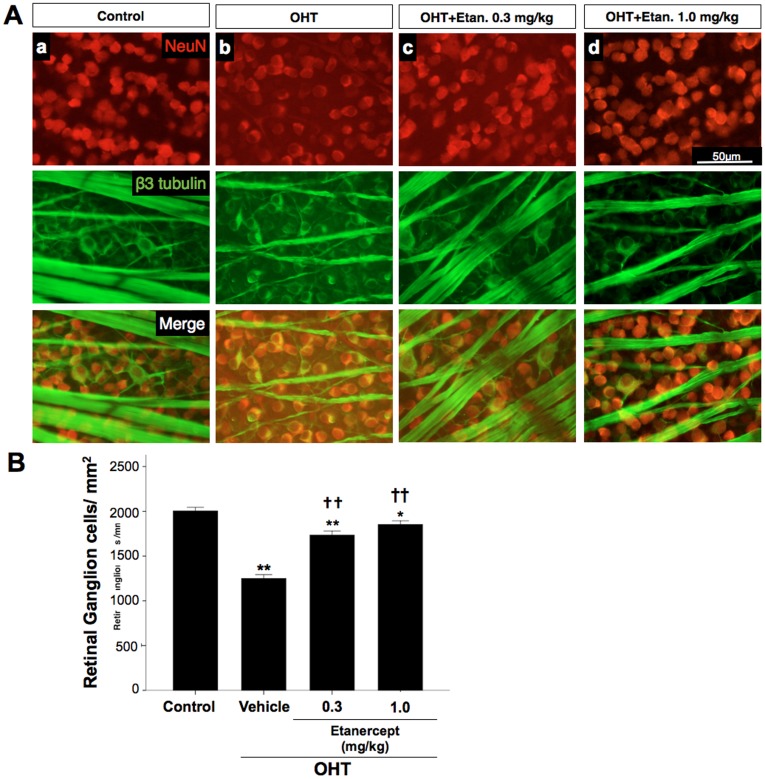
Etanercept prevents the loss of RGCs following OHT induction. **A**, Flat-mounted retina double immuno-labeled with TUJ-1 and NeuN antibodies to visualize RGCs and their axons. OHT resulted in a loss of RGCs (b) that was prevented with Etanercept (c, d). **B**, Bar graph showing quantitation of RGC survival. Data are expressed as mean ± SEM; n = 10 at each time point. Kruskal-Wallis test, **P<0.01 comparing three groups (A), Mann-Whitney U test, *P<0.05, **P<0.01 comparing sham-operated control and vehicle-treated OHT; ^††^P<0.01 comparing OHT treated with vehicle vs. Etanercept. NeuN (red), β-tubulin (green), Original magnification: ×20; Scale bar, 50 µm. OHT, ocular hypertension; Etan., Etanercept.

We next investigated whether elevation of IOP leads to the hallmark feature of glaucoma, a loss of RGCs, in the current model [26]–[28]. In the sham-operated control group, the number of RGCs visualized in retinal whole-mounts (Fig. 8A) that were co-labeled with both TUJ1 and NeuN was 2004.4±40.4 per mm^2^, whereas the number in OHT retinas treated only with saline was 38% lower (Fig. 8B, Mann-Whitney U test, P<0.001). Etanercept at either 0.3 or 1.0 mg/kg prevented this decrease and maintained the number of RGC at near-normal levels (Fig. 8B, Mann-Whitney U test, P<0.001 for either dosage compared to saline-treated OHT controls).

## Discussion

The current study confirms and extends previous findings linking retinal ganglion cell (RGC) loss in glaucoma to TNF-α. Our results demonstrate this link using a different method to induce ocular hypertension (OHT) and a different species than our earlier study, while for the first time identifying microglia at the optic nerve head (ONH) as the source of TNF-α. In addition, we show that Etanercept (Enbrel®), a widely used TNF-α antagonist, attenuates inflammation and RGC loss.

In the present study, we elevated intraocular pressure (IOP) by episcleral vein cauterization (EVC), which led to axon degeneration in the optic nerve and eventual loss of RGCs. Elevated IOP caused levels of TNF-α to increase within 3 days, and by 7 days, levels were 17-fold higher than normal and remained elevated for at least 4 weeks. However, compared to proinflammatory eye disease such as experimental uveitis induced by lipopolysaccharide [29], the level of TNF-α is much lower in our model. Nevertheless, along with the increases of TNF-α in our studies, we observed a massive expansion of the macrophage/microglial population around the ONH after 5–7 days. This change is consistent with our previous proposal that the cytotoxic effect of OHT-induced TNF-α might be mediated by CD11b+ microglia in a mouse model of chronic OHT [20]. However, the previous study left several major questions unanswered. One limitation of that study was the use of laser-induced angle closure to elevate OHT, which could have caused an inflammatory response and an increase in proinflammatory cytokines, including TNF-α, as a result of 100 confluent laser spots on the corneal limbus. By using three-point episcleral vein cauterization, we minimized the inflammatory effect associated with the model itself. Secondly, in the previous study we investigated the role of TNF-α using genetically altered mouse strains and immunodepletion. The present study brings us closer to clinical translation by showing that Etanercept, an agent that is already in wide clinical use for other indications, is effective in rescuing RGCs from OHT-induced death. Since maintaining elevated IOP is crucial in any model of glaucoma, Etanercept was given systemically rather than intravitreally, and did not affect IOP (Fig. 1). Another technical issue is that cauterizing the episcleral vein may lead to retinal ischemia, which can also result in RGC death [30], [31]. Ischemia and hypoxia have been implicated in the development of RGC death in patients with glaucoma [18], [32], and thus the model of chronic OHT used here may mimic multiple features of the clinical disorder.

Retinal microglia are resident macrophages derived from myeloid progenitor cells that are capable of phagocytic activity in multiple conditions [33], [34] that include aging, hypoxia, neurodegeneration, and injury. In all of these instances, microglia transform from a resting state with a ramified morphology to an activated phenotype [35] that retract their processes and exhibit a rounded cell body. Iba-1 antibody is a microglial/macrophage marker that is expressed in both the resting and activated states [36]. Immunohistochemical staining on whole-mounts showed increased numbers of amoeboid, activated Iba-1-positive microglia gathering toward the ONH after OHT induction, in contrast to the mosaic distribution of ramified “resting” microglia seen in sham-operated control retinas (Figs. 3A, 4A). It is possible that elevated IOP with subsequent hypoxia may break down the blood-retina-barrier at the ONH [37]–[39] or cause local stress at the ONH [40], eliciting a strong inflammatory and glial response. Our colocalization studies indicate that Iba-1-positive microglia located around the ONH are the likely source of TNF-α seen after OHT (Fig. 4D–F). In Alzheimer’s disease (AD), microglia are activated at sites of neuronal degeneration [41]. Co-culture of amyloid β protein with activated microglia produces TNF-α [42] and chronic neuronal TNF-α expression in an AD mouse model, and results in neuronal cell death [43], suggesting that microglia are the source of TNF-α. However, the source of TNF-α in the eye has been controversial. Although it is widely acknowledged that increased IOP can trigger an inflammatory response involving the ONH area [11], [20], [44], various reports have suggested that glial cells [8], [45], astrocytes [46] and Müller cells [47] are the source of TNF-α. Here, OHT induction resulted in extensive microglia and astrocyte activation as visualized with Iba-1 and GFAP staining, respectively. However, TNF-α colocalized only with microglia, and not with either astrocytes or Müller cells (data not shown) 5 days after EVC. Since TNF-α was seen to be elevated from day 3 onwards after OHT induction, it is possible that astrocytes and Müller cells may express cytokines and chemokines at an earlier point that lead to microglial activation and further enhancement of the neural injury. Microglial activation and clustering at the ONH and central retina have been shown to be early events, rather than late consequences, of RGC degeneration in another animal model of glaucoma [48]. The morphology of Iba-1 positive microglia changes to an activated phenotype as early as 3 days after IOP elevation [49], and our results of elevated expression of TNF-α on or before day 3 (Fig. 2B) implicates microglia as the major source of TNF-α expression after OHT. This is also supported by studies by Kreutz et al. [50] who demonstrated that in response to optic nerve crush injury, macrophages are recruited to the optic nerve and their phagocytic activity is accompanied by demyelination of RGC axons, which was abolished with the blockade of TNF-α signaling. Another recent study [51] showed that soluble TNF- α production from microglia is required for the death of co-cultured dopaminergic neurons, and that Etanercept could attenuate cell death without affecting other possible effects of microglia. In our previous study, we showed that the effect of TNF-α on RGC death is indirect and is dependent upon TNFR2-mediated activation of microglia [20]. In addition, other studies have shown that FasL, a TNF family member, is upregulated in microglia in the glaucomatous retina [52], and that the membrane-bound form of FasL acts as a key effector of RGC death downstream from TNF- α elevation [53].

Another finding in our study was the early dramatic decrease in retinal levels of NF-L and NF-M 14 days after OHT induction (Fig. 7), which preceded the loss of RCGs at day 28 (Fig. 8). These findings are consistent with earlier studies showing that axonal dysfunction precedes neuronal loss [54]. The effect of TNF-α inhibition in maintaining near-normal levels of neurofilament and RGCs in rats with OHT suggests that Etanercept may protect axons and secondarily RGC somata even in the presence of persistent ocular hypertension.

**Figure 9:**
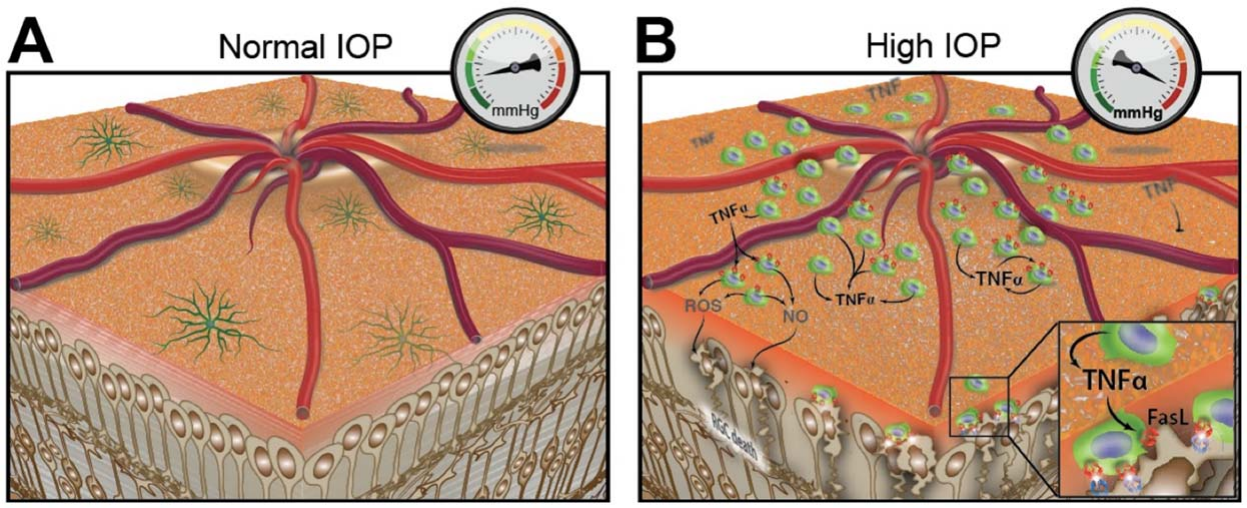
Schematic illustration of retinal ganglion cell (RGC) death following elevation of intraocular pressure (IOP). A, Ramified quiescent microglia in eyes with normal IOP. B, Elevated IOP leads to increased numbers of activated microglia with amoeboid morphology around the optic nerve head. These microglia appear to secrete TNF-α leading to RGC death. Other molecules, including FasL on microglia, nitric oxide (NO), and reactive oxygen species (ROS) may also play a role in RGC death.

In conclusion, our results suggest that elevation of the pro-inflammatory cytokine TNF-α and ONH inflammation play an important role in linking elevated intraocular pressure to the loss of optic nerve axons and RGCs that are the hallmark features of glaucoma (Fig. 9). Lowering of IOP is currently the most effective clinical approach to reducing the progression of glaucomatous visual loss [55], [56]. However, some patients continue to lose vision despite lowering of IOP, suggesting the need for adjunct treatments. The present results, supported by considerable evidence from other studies, suggest that Etanercept or other TNF- α antagonists or suppressors of inflammation should be considered as an adjunct therapy in the treatment of glaucoma.

## Materials and Methods

### Animals

All animal experiments followed the guidelines of the ARVO Statement for the Use of Animals in Ophthalmic and Vision Research and were approved by the Animal Care Committee of The Massachusetts Eye and Ear Infirmary. Male Brown Norway rats (8–10 weeks) were purchased from Charles River Laboratories (Wilmington, ME). A total of 140 rats between 200 and 300 g weight were used. Animals were housed under a 12-h light/dark cycle with free access to water and food. For general anesthesia, a mixture of ketamine (100 mg/kg; Phoenix Scientific, St. Joseph, MO) and xylazine (10 mg/kg; Phoenix Scientific) was administered intramuscularly.

### Experimental Ocular Hypertension

Chronic ocular hypertension (OHT) was induced in the right eye (total *n* = 140) by episcleral vein cauterization (EVC) according to published procedures with minor modifications [57], [58]. Briefly, after animals were deeply anesthetized, an incision was made through the conjunctiva and Tenon’s capsule on the limbal periphery. The episcleral veins in the right eye were identified by their location in relation to the extraocular muscles; three of them (two dorsal veins and one temporal ventral vein) were cauterized with a hand-held ophthalmic cautery (Electric Eye Cautery, Rumex International Co., St. Petersburg, FL) under a surgical microscope. The left eye received sham surgery (conjunctival incisions without cauterization). Successful OHT was defined as intraocular pressure (IOP) elevation greater than 30% of baseline IOP and no cauterization-associated complications after EVC.

### Measurement of Intraocular Pressure

IOP was measured in both eyes with the TonoLab tonometer TV02 (Helsinki, Finland) with rats under topical anesthesia with 0.5% proparacaine hydrochloride eye drops. IOP was measured before EVC, immediately after cauterization, and then on days 1, 7, 14, 21, and 28. The TonoLab acquires six valid rebounds of the device from the eye and takes the mean of the middle four readings to make one summary measurement. The instrument indicates the reliability of the reading by the position of a bar next to the reported value. A single set of readings was collected with the best reproducibility indicator (no bar) at each time point during the IOP measurements. Rats were excluded from further analysis if the peak IOP was <6 mm Hg above baseline or if the treated eye developed corneal ulceration, hyphema, or other ocular pathology. In total, 13 rats were excluded based on these criteria.

### Treatment with a Soluble TNF-α Receptor

Etanercept, a soluble 75 kDa TNF-α receptor/Fc fusion protein (Enbrel®, Amgen, Thousand Oaks, CA, USA), was reconstituted with sterile water according to the manufacturer’s instructions. Rats were separated into three groups. Starting one day after induction of chronic glaucoma, two groups (n = 10 each) were given subcutaneous injection of Etanercept at either 0.3 mg/kg or 1 mg/kg three times a week until the day of sacrifice. A third group (n = 10) received intraperitoneal injection of physiological saline three times a week. These doses were selected based upon previous studies that demonstrated the effectiveness of the drug in other disease models [59]–[61].

### Retinal Immunohistochemistry

For retinal cryosection immunostaining, sections through the retina (10 µm) with the optic nerve attached were pre-blocked (PBS containing 10% goat serum, 0.5% gelatin, 3% BSA, and 0.2% Tween-20) and then incubated with rabbit anti-Iba-1 antibody (1∶500; Wako Chemicals USA Inc., Richmond, VA) as a marker for microglia, anti-neurofilament light chain monoclonal antibody (1∶1000; Thermo Scientific), and/or anti-neurofilament middle chain monoclonal antibody (1∶1000; Cell Signaling Danvers, MA) overnight at 4°C. Alexa Fluor 488-conjugated goat anti-mouse IgG (Invitrogen, Carlsbad, CA) or Alexa Fluor 546-conjugated donkey anti rabbit IgG (Invitrogen) were used as secondary antibodies and incubated at room temperature for 1 hour prior to mounting in Vectashield mounting medium (Vector Laboratories, Burlingame, CA).

For whole-mount immunostaining, rat eyes were enucleated immediately after transcardiac perfusion. Post-fixed retinas were washed several times in fresh PBS, transferred to multi-well plates, and blocked in 10% normal goat serum (Sigma-Aldrich, St. Louis, MO) with 0.5% Triton X-100 in 0.1 ml PBS overnight. The retinas were then incubated in the same medium containing antibody TUJ1 (1∶500; mouse anti-neuronal class III β-Tubulin, Covance, Berkeley, CA), anti-NeuN (1∶500; made in mouse, clone A60, Chemicon, Temecula, CA), mouse monoclonal antibody to TNF-α (1∶200; Abcam, Cambridge, MA), and/or rabbit anti- Iba-1 antibody (1∶500; Wako Chemicals USA Inc., Richmond, VA) for 1 day at 4°C on a shaking platform. After three further washes, retinas were incubated with a goat antibody to mouse IgG conjugated to Alexa Fluor 546 and a donkey antibody to rabbit IgG conjugated to Alexa Fluor 488 diluted in blocking medium (1∶400; Invitrogen) overnight at 4°C and flat-mounted onto slides after making four radial slits and coverslipped in Vectashield mounting medium.

### Assessment of RGC Survival

Surviving RGCs were quantified in retinal whole mount preparations that were double-immunolabeled with the TUJ1 antibody directed against βIII tubulin and anti-NeuN. Within the ganglion cell layer, βIII tubulin is expressed only in RGCs, and thus immunostaining for this antigen can be used to distinguish RGCs from the many amacrine cells that are also in this layer [62], [63]. TUJ1- and NeuN-immunofluorescent cells in the ganglion cell layer were visualized with a Zeiss LSM 510 META laser scanning confocal microscope using a 20× objective and were counted manually in eight confocal fields in each quadrant of the retina using ImageJ software and the cell counter plugin. We counted four fields in the center near the ONH and four in the periphery, and then averaged the 32 counts for each retina to obtain RGCs per mm^2^.

### Assessment of Optic Nerve Axon Survival

RGC axons were quantified in the retrobulbar optic nerve 10 mm from the globe under light microscopy. At least three sections were used from each of six rats for each condition. Optic nerves were placed immediately into fixative consisting of 2.5% glutaraldehyde and 2% formaldehyde in 0.1 M cacodylate buffer with 0.08 M CaCl_2_ overnight at 4°C. The tissue was washed in 0.1 M cacodylate buffer and postfixed in 2% aqueous OsO_4_. Segments were dehydrated in graded alcohols and embedded in Epon. One-micron sections were cut and stained with 1% toluidine blue in 1% borate buffer. Five regions from each section were chosen for quantitation of axon density. In brief, 100× photomicrographs of each section were collected using an Olympus Povis AX70 microscope (Olympus Optical, Tokyo, Japan). Axons were counted semi-automatically with a method described previously [64]. The entire surface area of the nerve was represented in each sample and axon density was calculated as the mean across the random micrographs. Measurements of the cross-sectional area of each nerve were combined with axon density to estimate the total number of axons per nerve.

### Evaluating Microglial Activation

Using whole-mounted retinas, we classified the activation of Iba-1-positive microglia as described [25] using four stages. Stage 1 are resting microglia, which have rod-shaped somata with fine, ramified processes; Stage 2 are activated ramified microglia with elongated cell bodies and long, thicker processes; Stage 3 are amoeboid microglia with a round-shaped cell bodies and short, thick, stout processes; Stage 4 are phagocytic cells with round-shaped somata, vacuolated cytoplasm, and lacking any processes observed at the light microscopic level. In all whole-mounts, photos were taken at 8× magnification with the ONH in the center. The numbers of Iba-1 positive cells with the characteristics of stage 3 or 4 activation were counted manually in individual confocal fields using NIH ImageJ software and the cell counter plugin.

### Colocalization of TNF-α and Microglia

Retinal microglia and astrocytes were visualized in retinal whole-mounts by immunostaining with antibodies directed against Iba-1 and GFAP as previously described. The antibody to TNF-α was used for colocalization studies as specified in the manufacturer’s recommendations. Whole-mounts were visualized using a Leica SP5 confocal microscope with 10× and 63× objectives. The fluorescence of individual fluorochromes was captured separately in sequential mode (diode 405, argon 488, HeNe 543, HeNe 633) after optimization to reduce bleed-through between channels using Leica software. A stack of optical sections was taken at intervals of 0.16 µm.

### Western Blot Analysis

Retinas from experimental eyes with chronic OHT and control eyes were dissected from the RPE-choroid at designated time points after EVC. Samples were separated electrophoretically on NuPAGE 4–12% Bis-Tris Gels (Invitrogen) and transferred onto PVDF membrane (0.45 µm; Millipore, Billerica, MA). After blocking with 3% nonfat dried milk, membranes were incubated overnight with a primary antibody against TNF-α (1∶100, Cell Signaling, Danvers, MA), neurofilament light chain (monoclonal antibody, 1∶1000; Thermo Scientific), and neurofilament middle chain (monoclonal antibody, 1∶1000; Cell Signaling). The blotted membranes were then incubated for 30 min at room temperature with an HRP-conjugated secondary antibody directed against rabbit IgG (1∶10,000; Jackson ImmunoResearch). Immunoreactive bands were visualized by enhanced chemiluminescence (ECL) and exposed onto Fuji RX film (Fuji-Film, Tokyo, Japan).

### Co-immunoprecipitation

To investigate whether we could detect Etanercept in the rodent retina, immunoprecipitation (IP) was performed as previously described. [65] Briefly, C57BL/6 mice (n = 2) received a single intraperitoneal injection of 25 mg Etanercept and were euthanized 24 hours later, perfused with PBS, and retinas were isolated under a dissecting microscope. Control mice (n = 2) received an equal volume of normal saline (N/S). Retinas from each group were pooled in 0.5 ml chilled lysis buffer and homogenized with a tissue grinder. Supernatants were collected after centrifugation at 15,000 rpm for 10 min at 4°C. Protein concentrations were determined by Bio-Rad protein assay (Hercules, CA). Equal amounts (500 µg) of the retinal lysates were incubated overnight at 4°C with 20 µl of protein A/G agarose beads (Protein A/G Plus; Pierce, Thermo Scientific). The beads were then washed seven times with chilled lysis buffer and immunopellets were separated by SDS-PAGE and transferred onto PVDF membranes, where they were probed overnight at 4°C with a mouse anti-human IgG1 Fc region antibody (R&D Systems). Membranes were then incubated with an HRP-conjugated donkey anti-mouse secondary antibody (Jackson Immunoresearch), developed with ECL, and exposed to Fuji RX autoradiographic film.

### ELISA Analysis for TNF-α

The tissue complex containing the vitreous and neural retina was collected at 3, 7, 14, 21, or 28 days after EVC. Proteins were extracted from each retina individually in 200 µl PBS containing a protease inhibitor mixture (Complete, Roche Diagnostics, Pleasanton, CA) and sonicated (10 W, 5 s, 4°C; Sonifier 250, Branson, Danbury, CT). The supernatant was collected after centrifugation at 14,000×g for 20 min at 4°C (Micromax RF, IEC, Needham Heights, MA), and the total protein concentration was measured with the DC protein assay kit (Bio-Rad, Hercules, CA). The level of TNF-α was determined with rat TNF-α enzyme-linked immunosorbent assay (ELISA) kits (R&D Systems) according to the manufacturer’s protocols with slight modification. TNF-α standards were reconstituted down to 1.5625 pg/ml. The absorbance at 450 nm was measured using a 96-well plate spectrophotometer (Spectramax 190, Molecular Devices, Sunnyvale, CA). Absorbance values were converted to TNF-α concentration by comparison with a simultaneously generated standard curve and then normalized by the level of protein per retina. Standard curves showed an R^2^ value >0.98, demonstrating the sensitivity and reliability of the method.

### Statistical Analysis

Results are expressed as mean values ± standard error of mean (SEM). Statistical analysis was performed nonparametrically using Mann-Whitney U-test and Kruskal-Wallis tests (SPSS Statistics 17.0, Chicago, IL). A p-value of less than 0.05 was considered statistically significant.

## Supporting Information

Figure S1 **Immunoprecipitation of Etanercept from retinal lysates.** Five hundred mg of retinal lysate was incubated with anti-human IgG1 antibody and protein A/G agarose beads. Etanercept and mouse IgG were used as a positive control. Mice treated with Etanercept showed immunoprecipitation of Etanercept, whereas control mice did not. OHT, ocular hypertension; Etan., Etanercept.
(TIF)
