# Supplementary figures and images for: Etanercept, a Widely Used Inhibitor of Tumor Necrosis Factor-α (TNF- α), Prevents Retinal Ganglion Cell Loss in a Rat Model of Glaucoma
Source: PLoS One. 2012 Jul 3;7(7):e40065. doi: 10.1371/journal.pone.0040065 (PMC3388998; doi:10.1371/journal.pone.0040065)

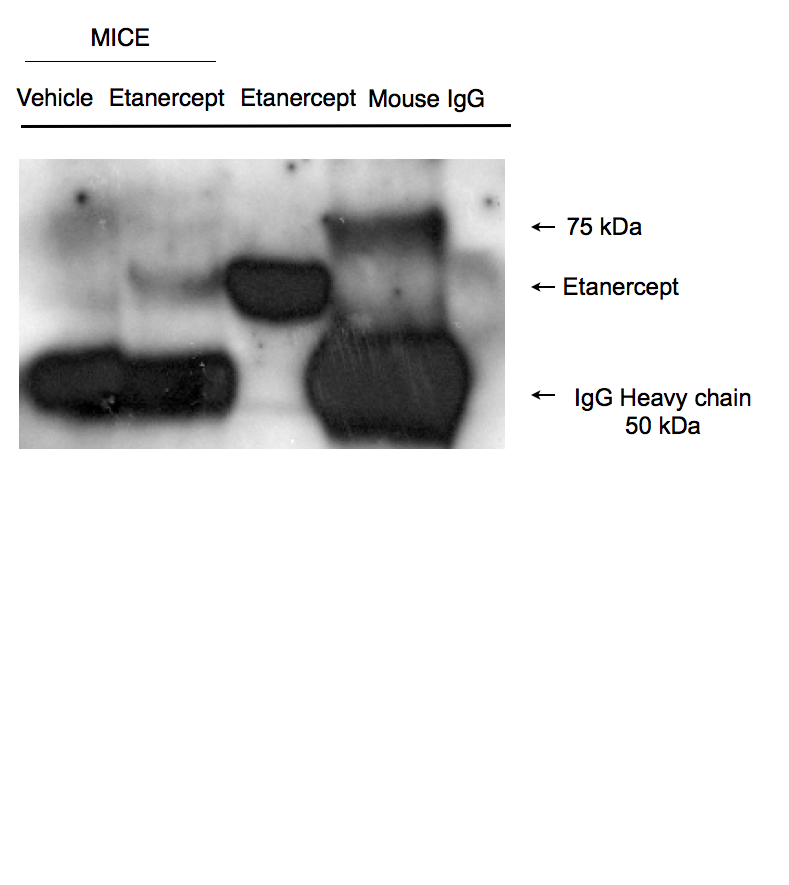

Supplement: Figure S1 — Immunoprecipitation of Etanercept from retinal lysates. Five hundred mg of retinal lysate was incubated with anti-human IgG1 antibody and protein A/G agarose beads. Etanercept and mouse IgG were used as a positive control. Mice treated with Etanercept showed immunoprecipitation of Etanercept, whereas control mice did not. OHT, ocular hypertension; Etan., Etanercept. (TIF) [file pone.0040065.s001.tif]
